# The stem cell growth factor receptor KIT is not expressed on interstitial cells in bladder

**DOI:** 10.1111/jcmm.13054

**Published:** 2016-12-20

**Authors:** Thomas Gevaert, Dirk De Ridder, Els Vanstreels, Dirk Daelemans, Wouter Everaerts, Frank Van Der Aa, Isabel Pintelon, Jean‐Pierre Timmermans, Tania Roskams, Clara Steiner, Jochen Neuhaus

**Affiliations:** ^1^ Laboratory of Experimental UrologyOrgan SystemsKU LeuvenLeuvenBelgium; ^2^ Translational Cell and Tissue ResearchDepartment of Imaging and PathologyKU LeuvenLeuvenBelgium; ^3^ Department of PathologyAZ KlinaBrasschaatBelgium; ^4^ Department of UrologyUZ LeuvenLeuvenBelgium; ^5^ Rega Institute for Medical ResearchKU LeuvenLeuvenBelgium; ^6^ Department of Veterinary SciencesUniversity of AntwerpAntwerpBelgium; ^7^ Klinik und Poliklinik für UrologieUniversity of LeipzigLeipzigGermany

**Keywords:** KIT, bladder, interstitial cells, immunohistochemistry, mast cells, immunofluorescence

## Abstract

The mast/stem cell growth factor receptor KIT has long been assumed to be a specific marker for interstitial cells of Cajal (ICC) in the bladder, with possible druggable perspectives. However, several authors have challenged the presence of KIT ^+^ ICC in recent years. The aim of this study was therefore to attempt to clarify the conflicting reports on KIT expression in the bladder of human beings, rat, mouse and guinea pig and to elucidate the possible role of antibody‐related issues and interspecies differences in this matter. Fresh samples were obtained from human, rat, mouse and guinea pig cystectomies and processed for single/double immunohistochemistry/immunofluorescence. Specific antibodies against KIT, mast cell tryptase (MCT), anoctamin‐1 (ANO1) and vimentin were used to characterize the cell types expressing KIT. Gut (jejunum) tissue was used as an external antibody control. Our results revealed KIT expression on mast cells but not on ICC in human, rat, mouse and guinea pig bladder. Parallel immunohistochemistry showed KIT expression on ICC in human, rat, mouse and guinea pig gut, which confirmed the selectivity of the KIT antibody clones. In conclusion, we have shown that KIT ^+^ cells in human, rat, mouse and guinea pig bladder are mast cells and not ICC. The present report is important as it opposes the idea that KIT ^+^ ICC are present in bladder. In this perspective, functional concepts of KIT ^+^ ICC being involved in sensory and/or motor aspects of bladder physiology should be revised.

## Introduction

There is a general consensus in the literature that the bladder contains two populations of stromal or interstitial cells (IC): IC in the lamina propria 1, 2, 3 (LP) (i.e. the area between the urothelium and detrusor smooth muscle) and IC in the detrusor 4 (inter‐ and intramuscular). Having been reported in bladders from different species including rat 5, guinea pig 4, 6, mouse 7 and human beings 8, these two IC populations appear to be a constant, species‐independent finding. Functional data on bladder IC are still limited, and the general hypothesis is that LP IC might be involved in non‐neural sensory signal transduction from the urothelium towards the detrusor muscle, while detrusor IC might have a role in pacemaking and/or transduction of pacemaking signals in the detrusor area 3.

One of the major research topics for bladder IC has been the search for specific cell markers. Of the several markers reported in the literature, the mast/stem cell growth factor receptor KIT (also known as the proto‐oncogene c‐kit or CD117) is one of the most extensively studied ones. KIT is a tyrosine‐protein kinase which acts as a cell‐surface receptor for the cytokine KIT ligand/stem cell factor (SCF) and plays an essential role in the regulation of cell survival and proliferation, haematopoiesis, stem cell maintenance, gametogenesis, mast cell development, migration and function, and melanogenesis 9, 10. The inspiration to look for the presence of KIT in the bladder came from functional gut research, where KIT^+^ ICC are well known to play central roles in pacemaking and signal transduction 11. KIT was first described in the bladder of guinea pig by the group of McCloskey *et al*. 12 and has later also been detected in rat 13, mouse 14, guinea pig 15 and human bladder 16.

The notion that KIT is expressed on IC in the bladder has been challenged in recent years by several authors 6, 8, 17, 18, 19, 20, who presented evidence that KIT in the bladder might instead be expressed by mast cells (known to be KIT^+^). This has led to a lively debate on whether interspecies differences and antibody‐related issues might account for the discrepant findings of KIT expression in the bladder. We have been working on bladder IC for many years, and this ongoing debate inspired us to set up a comparative study focussing on KIT expression in the bladder of different species. The major aim of this study was to attempt to clarify the conflicting reports in the literature on KIT expression in the bladder and to elucidate the possible role of antibody‐related issues and interspecies differences in this matter. To this end, we used solid antibody controls and performed sound co‐localization experiments, which we believe are needed to provide robust, reproducible data on KIT expression in the bladder.

## Materials and methods

### Tissue sampling and processing

The study protocol was in accordance with the EU guidelines and approved by the institution's ethical committees.

Human bladder tissue samples were obtained from cystectomies, performed for invasive bladder cancer, without previous neo‐adjuvant treatment (intravesical or systemic). Samples from six cystectomies (performed for bladder cancer, three females and three males) were used and fully processed in this study. All cystectomy specimens were received freshly from the operation room on dry ice and processed within a time frame of 15 min. Bladders were opened from the anterior side, and full thickness bladder wall biopsies were taken from the bladder dome, as far as possible from the macroscopically visible tumour. Jejunum tissue samples (to serve as external positive antibody controls) were obtained from the tissue database of the institutional pathology department (KU Leuven).

Mice, rat and guinea pig bladder tissue were obtained from female and male adult animals (12 weeks, *n* = 3/sex type). Strains were Sprague Dawley rats, B6 mice and coloured BFA guinea pigs, housed in cages with full access to food and water. Animals were killed by cervical dislocation after isoflurane anaesthesia. Bladders were dissected, and the bladder dome was cut into two halves. A part of the jejunum was also dissected to use as external tissue control.

All bladder samples were immediately fixed in formalin 6% and subsequently embedded in paraffin. All biopsies were checked histologically for the presence of normal bladder tissue.

### Immunohistochemistry/immunofluorescence

Antibodies against KIT, mast cell tryptase (MCT), anoctamin‐1 (ANO‐1) and vimentin were selected for their specificities to the epitopes of the different species, as stated in the manufacturer's data sheets and as confirmed in the literature (Table 1). Some antibody clones showed reliable immunoreactivity in control tissue of all species, while the specificity of others was highly species‐dependent.

**Table 1:** Table listing the properties of the antibody clones used

Immunogen	Code	Clone	Manufacturer	Host	Target species	Titre IHC
KIT (peptide corresponding to amino acids 963 to 976 at the cytoplasmic C‐terminal part)	A4502	/	Dako, Glostrup, Denmark	Rabbit	h, m, r, gp	1/750
KIT (peptide corresponding to amino acids 23‐322 of human origin)	SC‐5535	H‐300	Santa Cruz Biotechnology, Dallas, US	Rabbit	h, m, r	1/100
MCT (human beings, not further specified)	M7052	AA1	Dako, Glostrup, Denmark	Mouse	h	Ready to use
MCT (peptide mapping within an internal region of MCT of mouse origin)	SC‐32474	G‐12	Santa Cruz Biotechnology, Dallas, US	Goat	m, r	1/100
Vimentin (synthetic peptide within human vimentin amino acid 400 to the C‐terminus (acetyl))	ab92547	EPR3776	Abcam, Cambridge, UK	Rabbit	h, m, r,	1/1000
Vimentin (pig eye lens, not further specified)	V6389	V9	Sigma‐Aldrich, Saint Louis, MO, USA	Mouse	gp	1/400
ANO‐1/DOG‐1 (part of the C‐terminus of the cytoplasmic domain of the human DOG‐1)	NCL‐L‐DOG‐1	K9	Leica Microsystems, Diegem, Belgium	Mouse	h	Ready to use
ANO‐1/DOG‐1 (Synthetic peptide of human TMEM16A protein)	Ab53212	/	Abcam, Cambridge, UK	Rabbit	h, m, r, gp	1/400

Immunofluorescence labelling was performed on 5‐μm sections. Sections were deparaffinized in xylene, followed by immersion in alcohol and rehydration. Before staining, heat‐induced epitope retrieval was performed (30 min. at 120°C in Bond Epitope Retrieval Solution 2 (Leica Biosystems, Belgium)). All stains consisted of a sequential approach: sections were incubated with the first primary antibody for 30 min. at room temperature, followed by the first secondary antibody during 30 min.; these steps were followed by the same cascade for the second set of primary and secondary antibodies. Each step was followed by a 3 × 5 min. wash in Bond Wash Buffer (Leica Biosystems). Before each incubation with primary antibody, slides were incubated with normal goat serum (diluted 1:5 in PBS) for 30 min. to block non‐specific epitopes. Nuclear counterstaining was performed with DAPI (300 nM in PBS). Secondary antibodies were Alexa 568 Goat anti‐mouse, Alexa 488 Goat anti‐mouse, Alexa 568 Goat anti‐rabbit and Alexa 488 Goat anti‐rabbit (Invitrogen, Life Technologies, Ghent, Belgium).

Images were collected with a Leica TCS SP5 laser scanning confocal microscope (Leica Microsystems, Mannheim, Germany), using a HCX PL APO 63.0× (NA:1.40) oil immersion lens. Different fluorochromes were detected sequentially using excitation lines of 405 nm (DAPI, blue colour), 488 nm (AlexaFluor488, green colour) or 561 nm (AlexaFluor568, red colour). Emission was detected between 410–480 nm, 493–555 nm and 566–630 nm, respectively. Overlap between red and green signal of comparable intensity visually results in yellow signal on the represented pictures.

IHC stains were performed with the automated Leica Bond‐Max system (Leica Microsystems, Belgium). The automated procedure consisted of blocking endogenous peroxidase activity using 0.3% H_2_O_2_ in methanol, heat‐induced antigen retrieval, incubation with primary antibodies for 15 min., incubation with a peroxidase‐labelled polymer during 30 min. and a subsequent incubation with a substrate–chromogen (mixed DAB refine) for 10 min. Nuclear counterstaining was performed with haematoxylin. Images were acquired using a Leica DM LB microscope equipped with a DC300FX camera (Leica Microsystems).

Negative controls consisted of omission of the primary antibody, resulting in absence of immunoreactivity. Positive controls consisted of specific and expected IHC reaction on external control tissue (jejunum): KIT positivity on ICC and mast cells, ANO‐1 positivity on ICC and MCT positivity on mast cells. Per species, bladder and jejunal tissue was invariably processed on the same glass slide to ensure identical staining conditions for target and control tissue.

## Results

**Figure 1:**
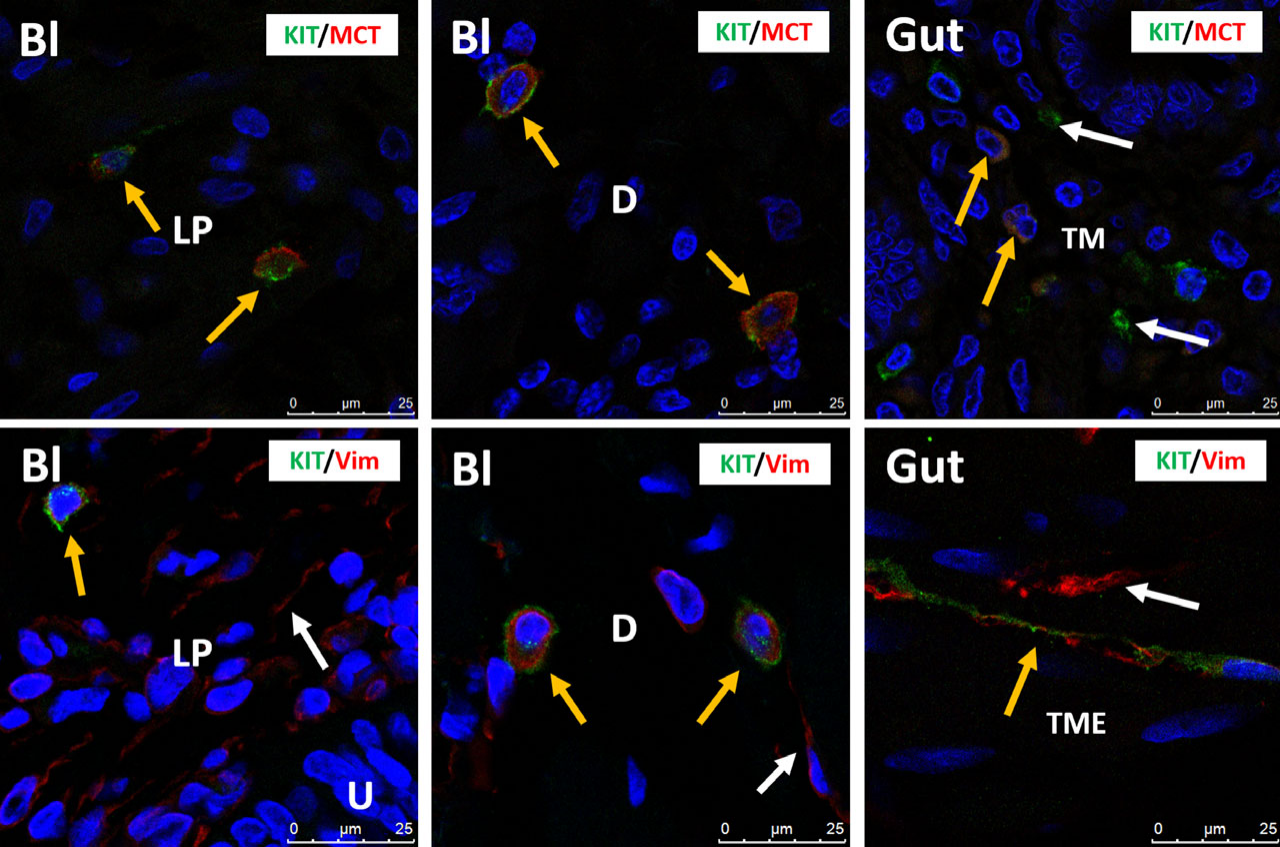
Upper panels: confocal immunofluorescence double staining for KIT (green) and MCT (red) showing co‐occurrence of both antigens on mast cells in the lamina propria and detrusor of human bladder (yellow arrows). Gut tissue is used as external tissue control, showing KIT ^+^/MCT ^+^ mast cells (yellow arrows), with some KIT ^+^/MCT ^−^ mast cells (white arrows) in the tunica mucosa (TM). Lower panels: confocal immunofluorescence double staining for KIT (green) and VIM (red) showing co‐occurrence of both antigens on mast cells in the lamina propria and detrusor of human bladder (yellow arrows). White arrows indicate the many VIM ^+^/KIT ^−^ IC. Gut tissue is used as external tissue control, showing co‐occurrence of both antigens on ICC in the tunica muscularis externa (TME) (yellow arrows). White arrows indicate KIT ^−^/VIM ^+^ IC. Bl, bladder; LP, lamina propria; D, detrusor; TM, tunica mucosa; TME, tunica muscularis externa. DAPI blue stain illustrates nuclei. Scale bars: 25 μm.

**Figure 2:**
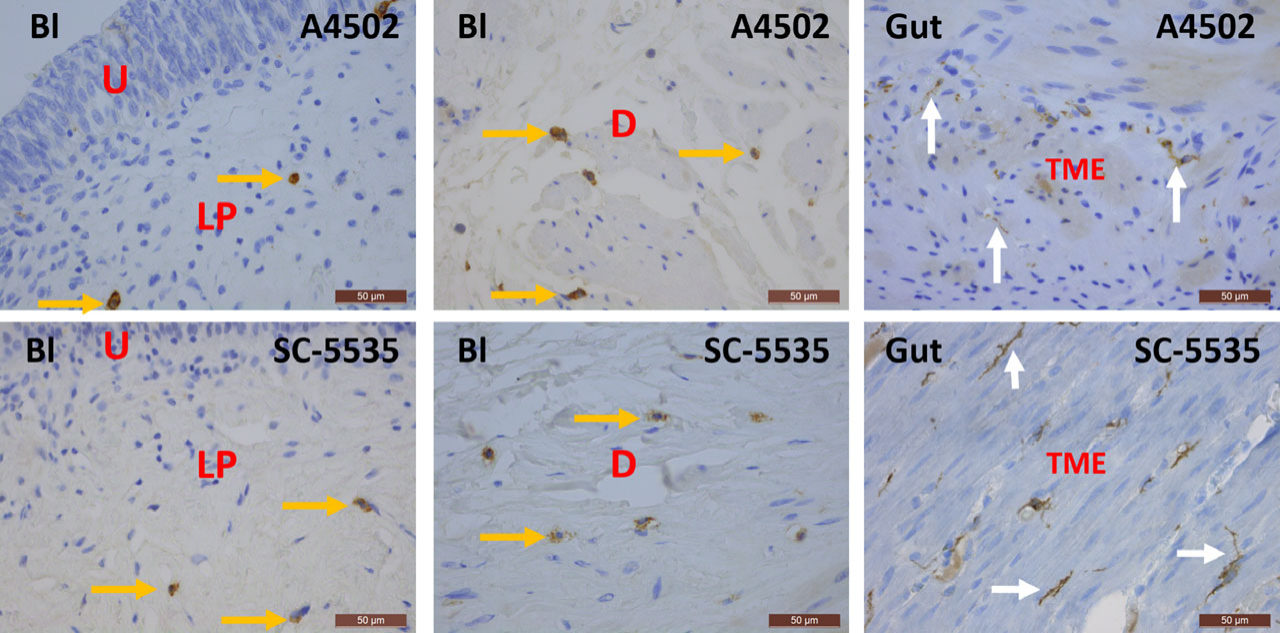
Immunohistochemical stains for KIT on human bladder and gut with clones A4502 (upper panels) and SC‐5535 (lower panels). In the bladder, KIT is observed on mast cells (yellow arrows), while in the tunica muscularis externa (TME) of the gut, KIT is found on ICC with long cytoplasmic processes (white arrows). The difference in morphology between these two cell types is remarkable. Both antibody clones show similar expression patterns for KIT in the bladder and gut. Bl, bladder; U, urothelium; LP, lamina propria; D, detrusor; TME, tunica muscularis externa. Scale bars: 50 μm.

**Figure 3:**
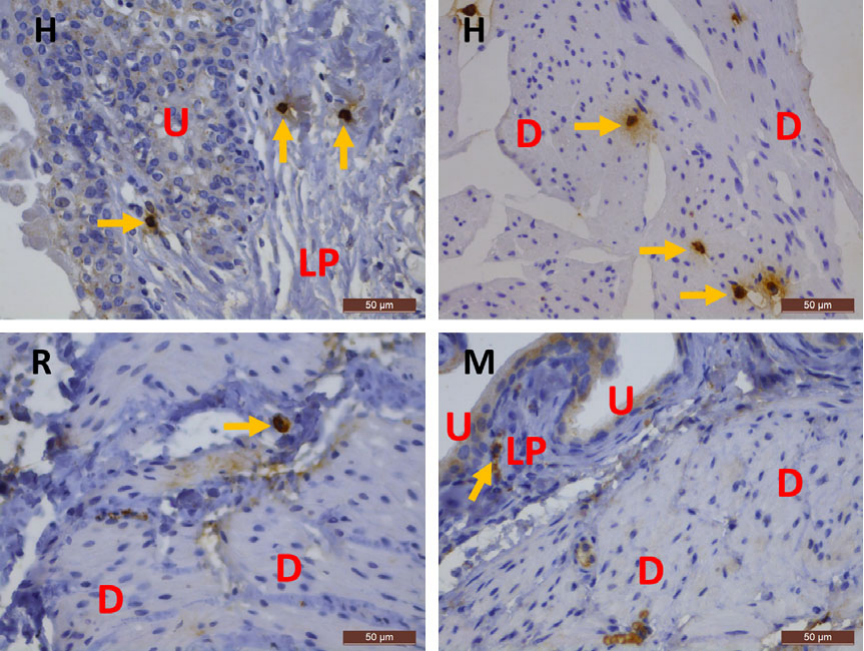
Immunohistochemical stains for MCT on the bladder in human beings (upper panels) and mouse and rat (lower panels). In human bladder, MCT ^+^ mast cells are frequently observed, while in mouse and rat bladder, mast cells are sparse (yellow arrows). H, human; R, rat; M, mouse; U, urothelium; LP, lamina propria; D, detrusor. Scale bars: 50 μm.

In human bladder, KIT was found on mast cells, but not on IC, as shown by the immunofluorescence (IF) co‐localization studies of KIT with MCT (Fig. 1). This observation was consistent throughout all bladder layers (LP and detrusor smooth muscle) and in all studied biopsies. A double staining of KIT with the broad mesenchymal marker VIM revealed that the typical long‐branched VIM‐positive IC were KIT^−^ (Fig. 1). Classic IHC stains for KIT and MCT showed similar distributions of both proteins on mast cells throughout the bladder wall (Figs 2, 3). Similar results were found with both KIT antibody clones (A4502 and SC‐5535) (Fig. 2). In the gut (jejunum), we observed KIT^+^/MCT^−^/VIM^+^ ICC in the tunica muscularis externa (TME) and KIT^+^/MCT^+^ mast cells in the tunica mucosa (TM), which confirms the specificity of the KIT antibody clones (Fig. 1). Classic IHC revealed a similar KIT expression pattern on jejunal ICC in the TME (similar results with both KIT antibody clones) (Fig. 2).

**Figure 4:**
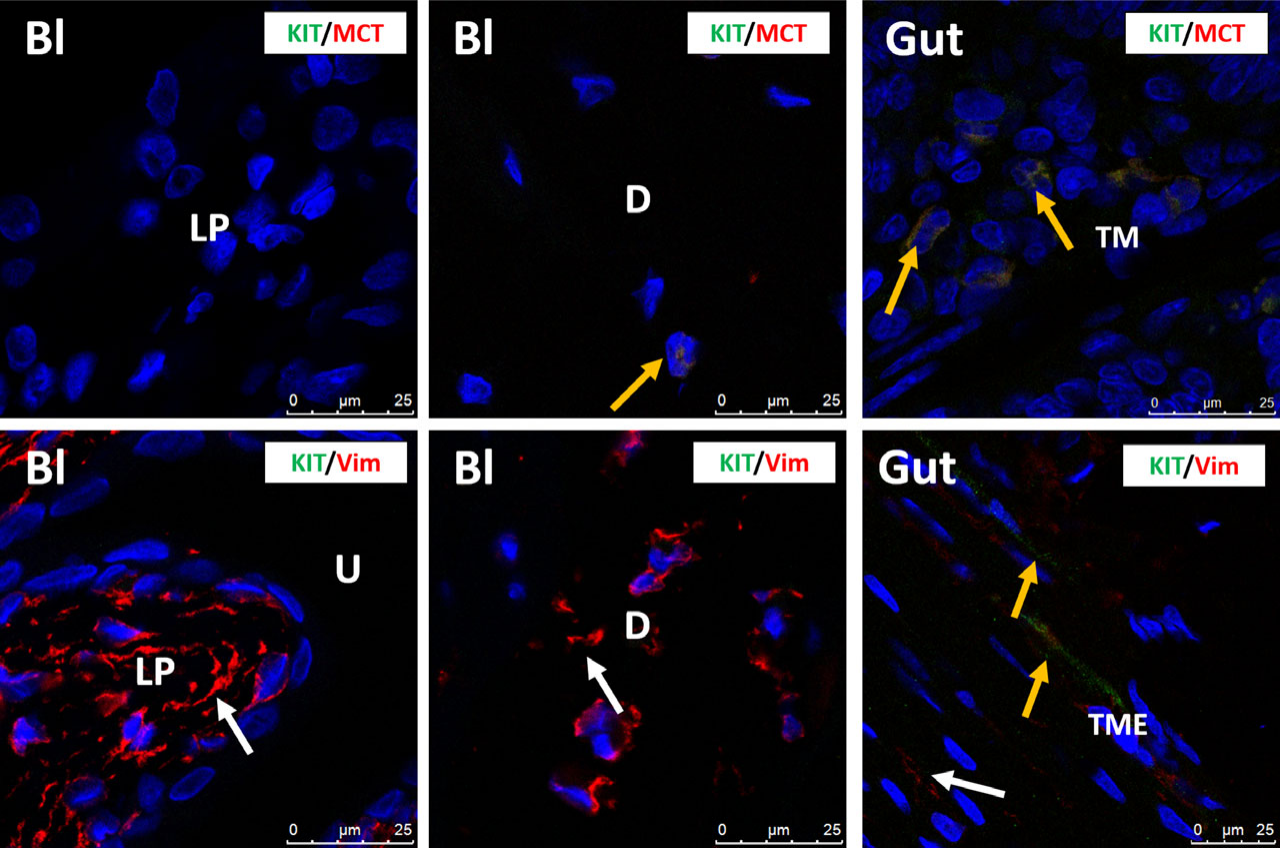
Upper panels: confocal immunofluorescence double staining for KIT (green) and MCT (red) showing co‐occurrence of both antigens on a mast cell in the detrusor of rat bladder (yellow arrow). Mast cells are very rare in rat bladder. Gut tissue is used as external tissue control, showing KIT ^+^/MCT ^+^ mast cells (yellow arrows) in the tunica mucosa (TM). Lower panels: confocal immunofluorescence double staining for KIT (green) and VIM (red) showing many VIM ^+^/KIT ^−^ IC (white arrows) in rat bladder. Gut tissue is used as external tissue control, with the presence of KIT ^+^/VIM ^+^ ICC (yellow arrows) and KIT ^−^/VIM ^+^ IC (white arrow) in the tunica muscularis externa (TME). Bl, bladder; LP,lamina propria; D, detrusor; TM,tunica mucosa; TME, tunica muscularis externa. DAPI blue stain illustrates nuclei. Scale bars: 25 μm.

**Figure 5:**
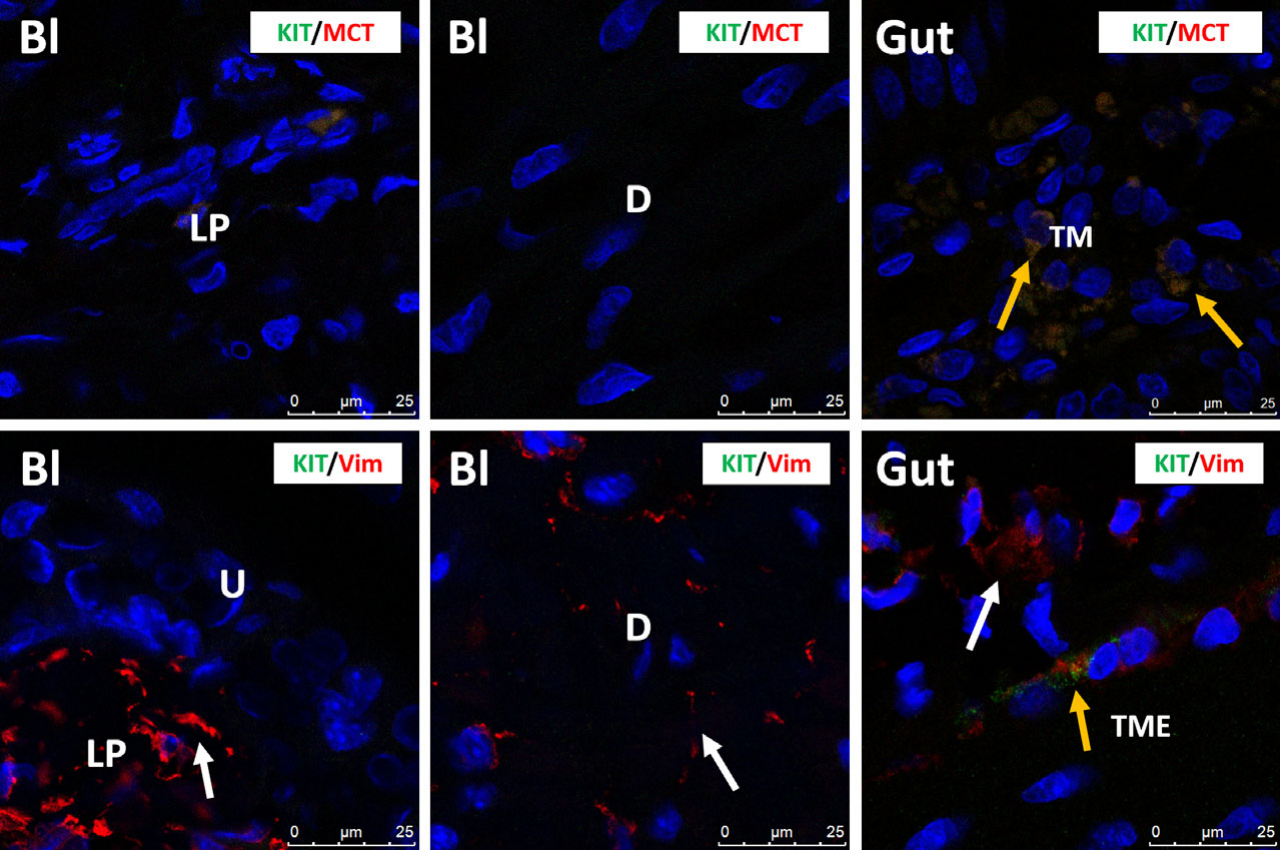
Upper panels: confocal immunofluorescence double staining for KIT (green) and MCT (red), showing no immunoreactive cells in mouse bladder. Mast cells are very rare in mouse bladder. Gut tissue is used as external tissue control, showing KIT ^+^/MCT ^+^ mast cells (yellow arrows) in the tunica mucosa (TM). Lower panels: confocal immunofluorescence double staining for KIT (green) and VIM (red) showing many VIM ^+^/KIT ^−^ IC (white arrows) in mouse bladder. Gut tissue is used as external tissue control, with the presence of KIT ^+^/VIM ^+^ ICC (yellow arrow) and KIT ^−^/VIM ^+^ IC (white arrow) in the tunica muscularis externa (TME). Bl, bladder; LP, lamina propria; D, detrusor; TM, tunica mucosa; TME, tunica muscularis externa. DAPI blue stain illustrates nuclei. Scale bars: 25 μm.

**Figure 6:**
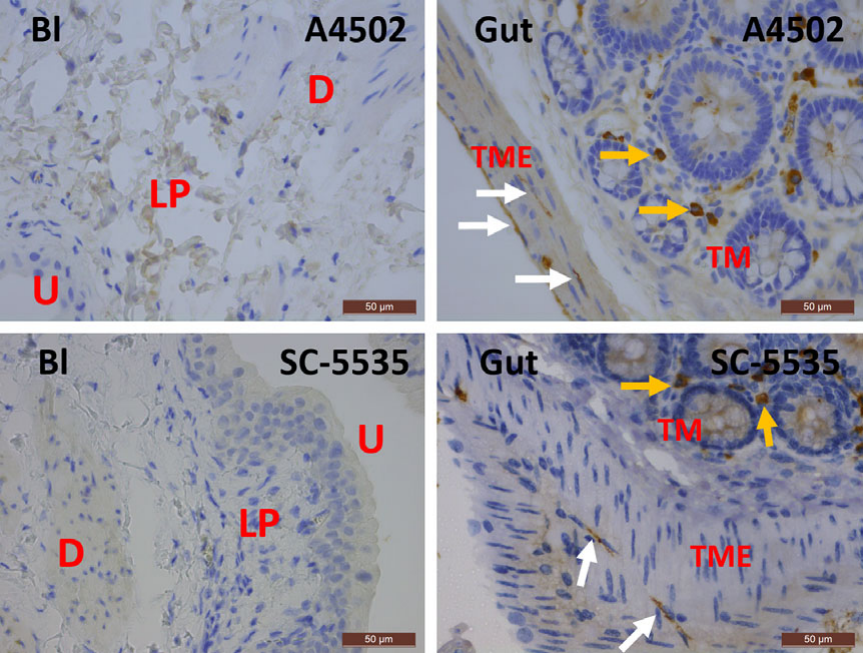
Immunohistochemical stains for KIT on rat bladder and gut with clones A4502 (upper panels) and SC‐5535 (lower panels). In the bladder, almost no KIT ^+^ mast cells are observed, while in the gut, KIT is found on mast cells (yellow arrows) and ICC with long cytoplasmic processes (white arrows). Both antibody clones show similar expression patterns for KIT in the bladder and gut. Bl, bladder; U, urothelium; LP, lamina propria; D, detrusor; TME, tunica muscularis externa; TM, tunica mucosa. Scale bars: 50 μm.

**Figure 7:**
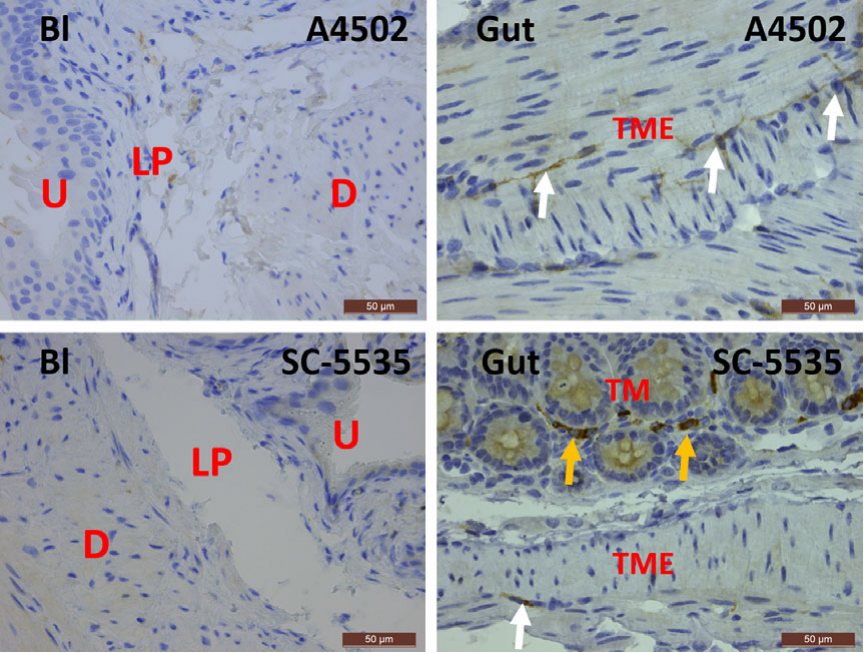
Immunohistochemical stains for KIT on mouse bladder and gut with clones A4502 (upper panels) and SC‐5535 (lower panels). In the bladder, almost no KIT ^+^ mast cells are observed, while in the gut, KIT is found on mast cells (yellow arrows) and ICC with long cytoplasmic processes (white arrows). Both antibody clones show similar expression patterns for KIT in the bladder and gut. Bl: bladder, U: urothelium, LP: lamina propria, D: detrusor, TME: tunica muscularis externa, TM: tunica mucosa. Scale bars: 50 μm.

In rat and mouse bladder, KIT was only observed on mast cells, which were occasionally present, and not on IC, as shown by the IF co‐localization of KIT with MCT (Figs 4, 5). This observation was consistent throughout all bladder layers and in all studied biopsies. Moreover, most studied rat and mouse bladder samples did not show any mast cells at all. A double staining of KIT with VIM showed all long‐branched VIM^+^ IC to be KIT^−^ (Figs 4, 5). Classic IHC stains for KIT and MCT in bladder yielded similar findings (i.e. only sporadically occurring mast cells showed co‐localization) (Figs 3, 6, 7). In the jejunum, we observed KIT^+^/MCT^−^/VIM^+^ ICC in the TME and KIT^+^/MCT^+^ mast cells in the TM, which confirms the specificity of the KIT antibody clones (Figs 4, 5). Classic IHC revealed a similar expression pattern of KIT on jejunal ICC in the TME (Figs 6, 7), with clone A4502 generating a better and finer distribution of ICC compared to clone SC‐5535.

**Figure 8:**
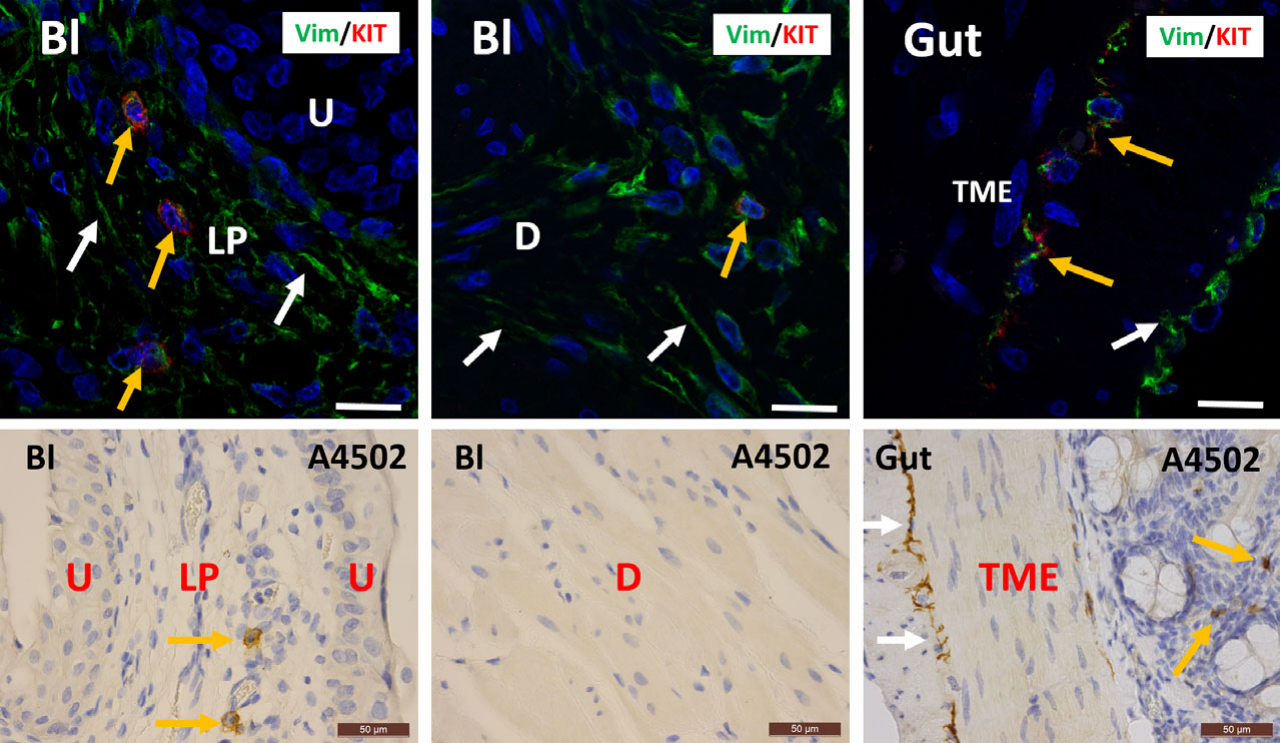
Upper panels: confocal immunofluorescence double staining for VIM (green) and KIT (red) showing co‐occurrence of both antigens on mast cells in the lamina propria and detrusor of guinea pig bladder (yellow arrows). White arrows indicate the many VIM ^+^/KIT ^−^ IC. Gut tissue is used as external tissue control, showing co‐occurrence of both antigens on ICC in the tunica muscularis externa (TME) (yellow arrows). White arrows indicate KIT ^−^/VIM ^+^ IC. Scale bars equal 25 μm. Lower panels: immunohistochemical stains for KIT on guinea pig bladder and gut with clone A4502. In the bladder, KIT is observed on mast cells (yellow arrows), while in the tunica muscularis externa (TME) of the gut, KIT is found on mast cells (yellow arrows) and ICC with long cytoplasmic processes (white arrows). Mast cells are rare in guinea pig bladder. The difference in morphology between these two cell types is remarkable. Scale bars equal 50 μm. Bl: bladder, U: urothelium, LP: lamina propria, D: detrusor, TME: tunica muscularis externa, TM: tunica mucosa.

In guinea pig bladder, we were unable to unequivocally confirm that KIT^+^ cells are mast cells due to the lack of reliable MCT/KIT double stains (we were unable to find decent MCT antibodies, to generate acceptable staining). However, we observed similar expression patterns for KIT as in other species, with only sparse KIT^+^ cells and with large populations of long‐branched VIM^+^/KIT^−^ IC in bladder LP and detrusor (Fig. 8). Furthermore, the granular staining pattern of KIT is compatible with mast cell origin. In gut tissue, we observed several KIT^+^/VIM^+^ long‐branched ICC in the TME (Fig. 8). Classic IHC stains for KIT in bladder yielded similar findings (i.e. only sporadically occurring mast cells in the LP and detrusor) (Fig. 8).

**Figure 9:**
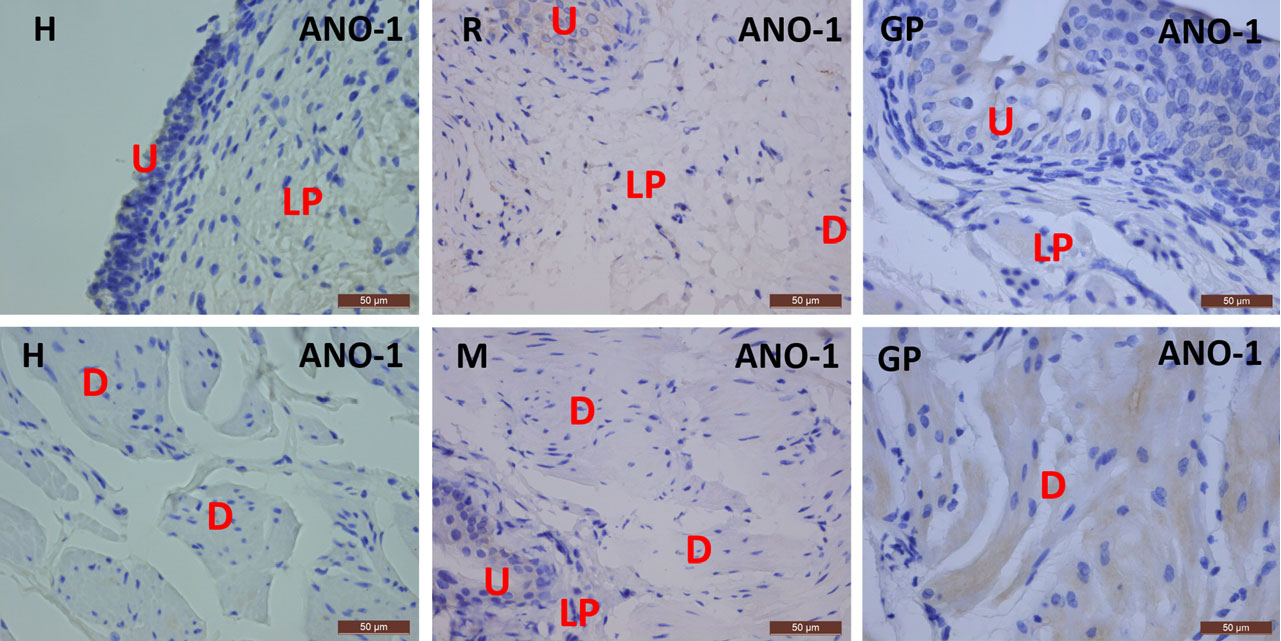
Immunohistochemical stains for ANO1 on human, rat, mouse and guinea pig bladder. No immunoreactivity was observed. U: urothelium, LP: lamina propria, D: detrusor. Scale bars: 50 μm.

**Figure 10:**
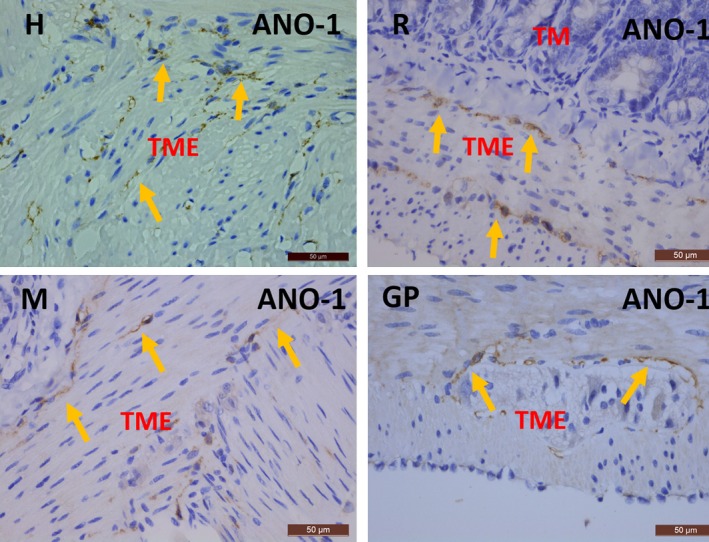
Immunohistochemical stains for ANO1 on human, rat, mouse and guinea pig gut. In all species, many ANO1^+^ ICC are seen in the TME (yellow arrows). TME: tunica muscularis externa, TM: tunica mucosa. Scale bars: 50 μm.

Classic IHC stains for the selective ICC marker ANO‐1 did not show any specific reaction in human, rat, mouse and guinea pig bladder (Fig. 9). Parallel IHC stains for ANO‐1 in human, rat, mouse and guinea pig gut clearly illustrated many ICC in the TME (Fig. 10).

## Discussion

KIT^+^ ICC have been studied in the gut for several decades, and there is now scientific consensus that KIT^+^ ICC in gut are involved in generating pacemaking signals and in signal transduction 11, 21. The discovery of KIT^+^ cells in the bladder was therefore a promising finding as it was assumed that these cells could have similar roles in the onset of spontaneous bladder contractions and in the transmission of electrical signals. In recent years, however, several authors have contested the hypothesis that the KIT^+^ cells in bladder are indeed ICC as seen in gut and instead argued that these cells could be mast cells 6, 8, 18, 19, 20 (which are also known to be KIT^+^). The present study provides ample evidence that KIT is expressed on mast cells and not on IC in human, rat, mouse and guinea pig bladder.

Several methodological issues might explain why KIT^+^ cells in the bladder have been (erroneously) considered as ICC in previous studies. Antibody selection is a first essential step towards optimal IHC results. Multiple comparative studies by the renowned IHC quality platform Nordiqc have shown great variability in specificity and reliability of different commercially available KIT antibody clones (www.nordiqc.com). Nordiqc recommends the A4502 clone used in the present study as one of the two preferential antibodies for the detection of KIT in human clinical diagnostics (www.nordiqc.org/Run-26-B7/Assessment/assessment-26-CD117). The use of one of the several other commercially available KIT antibody clones might have resulted in less specific reaction patterns. Antibody controls are a second key element for reliable IHC results, and one of the most rigorous antibody controls is the positive anatomical control, that is where the presence of the antigen in the specimen is known *a priori* and is not the target of the experimental treatment 22, 23. For KIT, gut tissue serves as an excellent positive antibody control, due to the well‐established presence of KIT^+^ ICC 24, 25. In this study, the specificity of the KIT antibodies was specifically validated by parallel staining of gut tissue on the same glass slide, which typically yielded KIT^+^ ICC (as further confirmed with double stains) and KIT^+^ mast cells. We did not find reports of the use of positive anatomical controls in literature on KIT expression in bladder 7, 12, 13, 14, 15, 16, 26, 27 (see also Table 2), possibly explained by the recent nature of these evolutions in IHC quality control protocols 22, 23. A third essential methodological parameter to reliably determine KIT expression in the bladder relates to the use of double stains. KIT is known to be expressed on several other cell types apart from ICC, that is mast cells, hematopoietic cells, spermatogonia and melanocytic cells 21. Of these populations, only mast cells are known to be expressed in the bladder, which is why we performed double stains with MCT in an attempt to rule out the possibility of a KIT^+^ mast cell population. KIT^+^ mast cells might have been wrongly interpreted as ICC due to the often similar morphological appearance of both cell types and the lack of reliable MCT/KIT double stains. The higher number of mast cells in human bladder compared to laboratory animals is remarkable, but might be explained by an inevitable selection bias: human bladder samples are likely to have at least some degree of inflammation (as reflected by a higher number of mast cells) as human cystectomies are, by definition, performed in a pathological setting, while cystectomies on animals can be performed under well‐controlled conditions without any relevant inflammation. In guinea pig bladder, we were unable to perform decent MCT/KIT double stains, due to the absence of reliable commercially available MCT antibodies. Most publications reporting on KIT^+^ ICC in bladder did not perform MCT/KIT double stains, with no reports of MCT/KIT double stains in guinea pig bladder 7, 12, 13, 15, 16, 27 (for an overview, see also Table 2).

**Table 2:** Overview of the properties of KIT antibodies used in literature reporting on KIT+ ICC in bladder

Study reference	Target	Manufacturer	Clone	Titre	Host	Gut tissue control	MCT double stain
McCloskey *et al*. 2002 12	Guinea pig	Gibco BRL, Grand Island, New York	N/A	1/200	Rat	Not included	Not included
Van Der Aa *et al*. 2004 27	Human beings	Dako, Glostrup, Denmark	A4502	N/A	Rabbit	Not included	Not included
Shafik *et al*. 2004 16	Human beings	Oncogene Research P, Cambridge, MA	N/A	1/100	Rabbit	Not included	Not included
Kubota *et al*. 2008 15	Guinea pig	Santa Cruz, Santa Cruz, CA	N/A	1/100	Goat	Not included	Not included
Johnston *et al*., 2010 26	Human beings	Santa Cruz, Santa Cruz, CA	N/A	1/200	N/A	Not included	Included
Kim *et al*. 2011^13^	Rat	R&D Systems, Minneapolis, MN	N/A	N/A	N/A	Not included	Not included
Yu *et al*. 2012 14	Mouse	Assay Biotechnology, Sunnyvale, CA	P‐10721	1/50‐1/500	Rabbit	Not included	Included
Monaghan *et al*., 2012 7	Human beings, Guinea pig	Dako, Glostrup, Denmark	A4502	1/1000	Rabbit	Not included	Not included

Another important element was the absence of expression of the ICC‐selective marker ANO‐1 28 (also known as DOG1 or TMEM16A) in human, rat, mouse and guinea pig bladder, which further favours the absence of typical ICC in bladder (ANO1‐IHC was validated by presence of ANO1^+^ ICC in gut). Several authors also reported on the absence of the typical ultrastructural ICC characteristics in IC in the bladder, lending additional evidence for another phenotype for these cells 8, 18, 19. We know from previous work that IC in the bladder constitute a complex cell family including fibroblasts, myofibroblasts, ICC‐like cells and telocytes, with different IC phenotypes in the lamina propria versus the detrusor 8, 18, 19. At present, our knowledge of IC in the bladder is rather limited, which is partially explained by the lack of specific cell markers, needed to selectively target these cells. As KIT has long been assumed to be present on bladder IC, several groups have tried to study the role of KIT^+^ IC in bladder physiology by blocking the KIT receptor with the KIT inhibitor imatinib mesylate (IM). Acute administration of IM caused decreased contractility in guinea pig and human bladder 29, while chronic administration of IM in neonatal rats resulted in impaired development of networks of intermuscular IC 5. Considering that IM also blocks PDGFRα 30, a receptor widely expressed on bladder IC 7, 20, we think that the effects of IM on bladder contractility and IC development might be explained *via* the PDGFRα pathway.

## Conclusions

In conclusion, we have shown that KIT^+^ cells in human, rat, mouse and guinea pig bladder are mast cells and not ICC. The present report is important as it opposes the idea that KIT^+^ ICC are present in bladder. In this perspective, functional concepts of KIT^+^ ICC being involved in sensory and/or motor aspects of bladder physiology should be revised. One of the major challenges in IC research will be to search for specific cell markers to investigate these cells more selectively.

## Conflict of interest statement

The authors confirm that there is no conflict of interest.

## Authors’ contributions

TG, IP, EV, CS, FVDA and WE performed the research. TG, DDR, DD and JN designed the research study. IP, JPT, WE, DD, EV and TR contributed essential reagents or tools. TG, CS, DDR and JN analysed the data. TG, DDR, CS, FVDA and JN wrote the manuscript.
